# A tutorial on methodological studies: the what, when, how and why

**DOI:** 10.1186/s12874-020-01107-7

**Published:** 2020-09-07

**Authors:** Lawrence Mbuagbaw, Daeria O. Lawson, Livia Puljak, David B. Allison, Lehana Thabane

**Affiliations:** 1 Department of Health Research Methods, Evidence and Impact, McMaster University, Hamilton, ON Canada; 2 Biostatistics Unit/FSORC, 50 Charlton Avenue East, St Joseph’s Healthcare—Hamilton, 3rd Floor Martha Wing, Room H321, Hamilton, Ontario L8N 4A6 Canada; 3 Centre for the Development of Best Practices in Health, Yaoundé, Cameroon; 4 Center for Evidence-Based Medicine and Health Care, Catholic University of Croatia, Ilica 242, 10000 Zagreb, Croatia; 5 Department of Epidemiology and Biostatistics, School of Public Health – Bloomington, Indiana University, Bloomington, IN 47405 USA; 6 Departments of Paediatrics and Anaesthesia, McMaster University, Hamilton, ON Canada; 7 Centre for Evaluation of Medicine, St. Joseph’s Healthcare-Hamilton, Hamilton, ON Canada; 8 Population Health Research Institute, Hamilton Health Sciences, Hamilton, ON Canada

**Keywords:** Methodological study, Meta-epidemiology, Research methods, Research-on-research

## Abstract

**Background:**

Methodological studies – studies that evaluate the design, analysis or reporting of other research-related reports – play an important role in health research. They help to highlight issues in the conduct of research with the aim of improving health research methodology, and ultimately reducing research waste.

**Main body:**

We provide an overview of some of the key aspects of methodological studies such as what they are, and when, how and why they are done. We adopt a “frequently asked questions” format to facilitate reading this paper and provide multiple examples to help guide researchers interested in conducting methodological studies. Some of the topics addressed include: is it necessary to publish a study protocol? How to select relevant research reports and databases for a methodological study? What approaches to data extraction and statistical analysis should be considered when conducting a methodological study? What are potential threats to validity and is there a way to appraise the quality of methodological studies?

**Conclusion:**

Appropriate reflection and application of basic principles of epidemiology and biostatistics are required in the design and analysis of methodological studies. This paper provides an introduction for further discussion about the conduct of methodological studies.

## Background

The field of meta-research (or research-on-research) has proliferated in recent years in response to issues with research quality and conduct [1–3]. As the name suggests, this field targets issues with research design, conduct, analysis and reporting. Various types of research reports are often examined as the unit of analysis in these studies (e.g. abstracts, full manuscripts, trial registry entries). Like many other novel fields of research, meta-research has seen a proliferation of use before the development of reporting guidance. For example, this was the case with randomized trials for which risk of bias tools and reporting guidelines were only developed much later – after many trials had been published and noted to have limitations [4, 5]; and for systematic reviews as well [6–8]. However, in the absence of formal guidance, studies that report on research differ substantially in how they are named, conducted and reported [9, 10]. This creates challenges in identifying, summarizing and comparing them. In this tutorial paper, we will use the term *methodological study* to refer to any study that reports on the design, conduct, analysis or reporting of primary or secondary research-related reports (such as trial registry entries and conference abstracts).

**Fig. 1:**
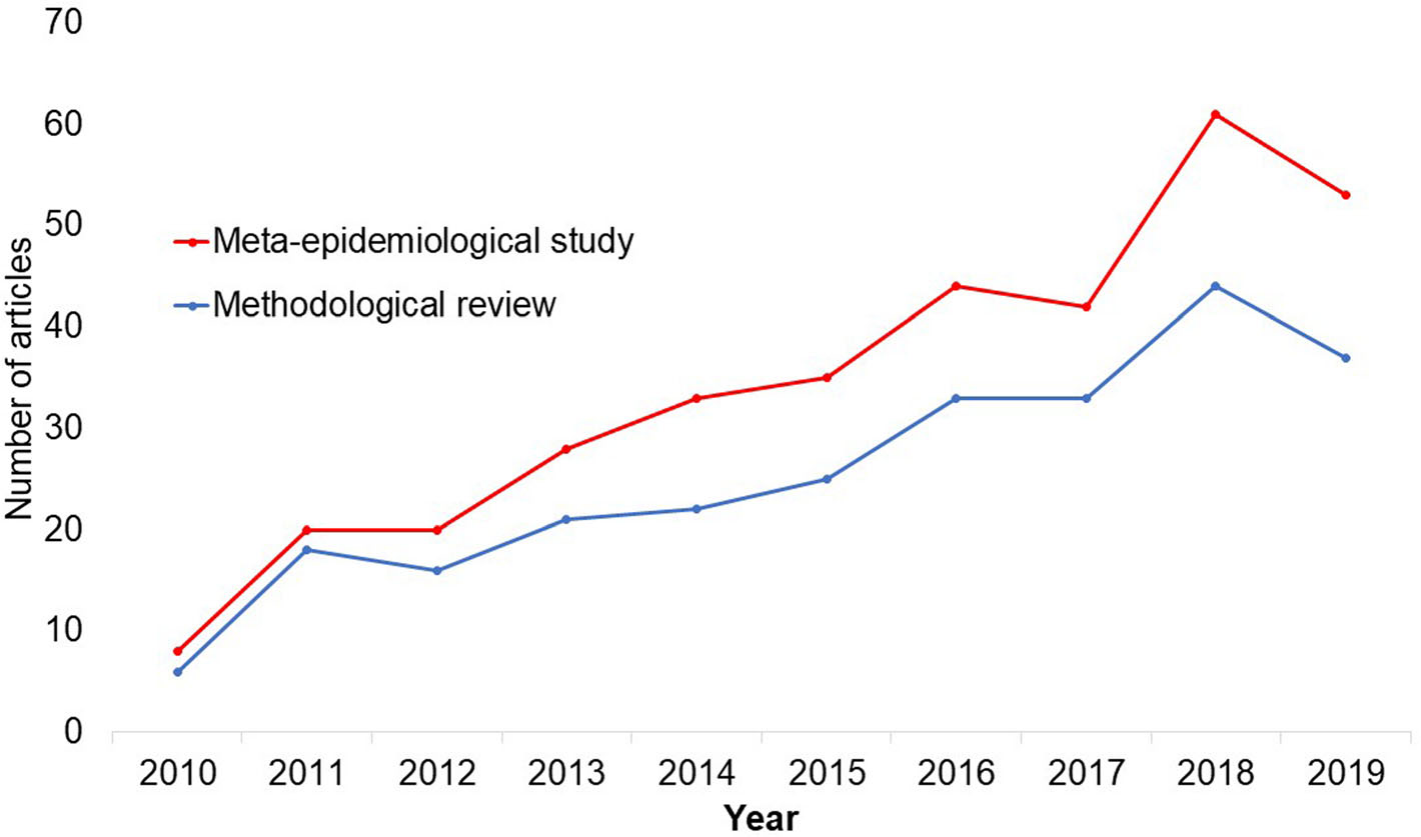
Trends in the number studies that mention “methodological review” or “meta- epidemiological study” in PubMed.

In the past 10 years, there has been an increase in the use of terms related to methodological studies (based on records retrieved with a keyword search [in the title and abstract] for “methodological review” and “meta-epidemiological study” in PubMed up to December 2019), suggesting that these studies may be appearing more frequently in the literature. See Fig. 1.

The methods used in many methodological studies have been borrowed from systematic and scoping reviews. This practice has influenced the direction of the field, with many methodological studies including searches of electronic databases, screening of records, duplicate data extraction and assessments of risk of bias in the included studies. However, the research questions posed in methodological studies do not always require the approaches listed above, and guidance is needed on when and how to apply these methods to a methodological study. Even though methodological studies can be conducted on qualitative or mixed methods research, this paper focuses on and draws examples exclusively from quantitative research.

The objectives of this paper are to provide some insights on how to conduct methodological studies so that there is greater consistency between the research questions posed, and the design, analysis and reporting of findings. We provide multiple examples to illustrate concepts and a proposed framework for categorizing methodological studies in quantitative research.

## Main text

### What is a methodological study?

Any study that describes or analyzes methods (design, conduct, analysis or reporting) in published (or unpublished) literature is a methodological study. Consequently, the scope of methodological studies is quite extensive and includes, but is not limited to, topics as diverse as: research question formulation [11]; adherence to reporting guidelines [12–14] and consistency in reporting [15]; approaches to study analysis [16]; investigating the credibility of analyses [17]; and studies that synthesize these methodological studies [18]. While the nomenclature of methodological studies is not uniform, the intents and purposes of these studies remain fairly consistent – to describe or analyze methods in primary or secondary studies. As such, methodological studies may also be classified as a subtype of observational studies.

Parallel to this are experimental studies that compare different methods. Even though they play an important role in informing optimal research methods, experimental methodological studies are beyond the scope of this paper. Examples of such studies include the randomized trials by Buscemi et al., comparing single data extraction to double data extraction [19], and Carrasco-Labra et al., comparing approaches to presenting findings in Grading of Recommendations, Assessment, Development and Evaluations (GRADE) summary of findings tables [20]. In these studies, the unit of analysis is the person or groups of individuals applying the methods. We also direct readers to the Studies Within a Trial (SWAT) and Studies Within a Review (SWAR) programme operated through the Hub for Trials Methodology Research, for further reading as a potential useful resource for these types of experimental studies [21]. Lastly, this paper is not meant to inform the conduct of research using computational simulation and mathematical modeling for which some guidance already exists [22], or studies on the development of methods using consensus-based approaches.

### When should we conduct a methodological study?

Methodological studies occupy a unique niche in health research that allows them to inform methodological advances. Methodological studies should also be conducted as pre-cursors to reporting guideline development, as they provide an opportunity to understand current practices, and help to identify the need for guidance and gaps in methodological or reporting quality. For example, the development of the popular Preferred Reporting Items of Systematic reviews and Meta-Analyses (PRISMA) guidelines were preceded by methodological studies identifying poor reporting practices [23, 24]. In these instances, after the reporting guidelines are published, methodological studies can also be used to monitor uptake of the guidelines.

These studies can also be conducted to inform the state of the art for design, analysis and reporting practices across different types of health research fields, with the aim of improving research practices, and preventing or reducing research waste. For example, Samaan et al. conducted a scoping review of adherence to different reporting guidelines in health care literature [18]. Methodological studies can also be used to determine the factors associated with reporting practices. For example, Abbade et al. investigated journal characteristics associated with the use of the Participants, Intervention, Comparison, Outcome, Timeframe (PICOT) format in framing research questions in trials of venous ulcer disease [11].

### How often are methodological studies conducted?

There is no clear answer to this question. Based on a search of PubMed, the use of related terms (“methodological review” and “meta-epidemiological study”) – and therefore, the number of methodological studies – is on the rise. However, many other terms are used to describe methodological studies. There are also many studies that explore design, conduct, analysis or reporting of research reports, but that do not use any specific terms to describe or label their study design in terms of “methodology”. This diversity in nomenclature makes a census of methodological studies elusive. Appropriate terminology and key words for methodological studies are needed to facilitate improved accessibility for end-users.

### Why do we conduct methodological studies?

Methodological studies provide information on the design, conduct, analysis or reporting of primary and secondary research and can be used to appraise quality, quantity, completeness, accuracy and consistency of health research. These issues can be explored in specific fields, journals, databases, geographical regions and time periods. For example, Areia et al. explored the quality of reporting of endoscopic diagnostic studies in gastroenterology [25]; Knol et al. investigated the reporting of *p*-values in baseline tables in randomized trial published in high impact journals [26]; Chen et al. describe adherence to the Consolidated Standards of Reporting Trials (CONSORT) statement in Chinese Journals [27]; and Hopewell et al. describe the effect of editors’ implementation of CONSORT guidelines on reporting of abstracts over time [28]. Methodological studies provide useful information to researchers, clinicians, editors, publishers and users of health literature. As a result, these studies have been at the cornerstone of important methodological developments in the past two decades and have informed the development of many health research guidelines including the highly cited CONSORT statement [5].

### Where can we find methodological studies?

Methodological studies can be found in most common biomedical bibliographic databases (e.g. Embase, MEDLINE, PubMed, Web of Science). However, the biggest caveat is that methodological studies are hard to identify in the literature due to the wide variety of names used and the lack of comprehensive databases dedicated to them. A handful can be found in the Cochrane Library as “Cochrane Methodology Reviews”, but these studies only cover methodological issues related to systematic reviews. Previous attempts to catalogue all empirical studies of methods used in reviews were abandoned 10 years ago [29]. In other databases, a variety of search terms may be applied with different levels of sensitivity and specificity.

### Some frequently asked questions about methodological studies

In this section, we have outlined responses to questions that might help inform the conduct of methodological studies.

**Q: How should I select research reports for my methodological study?**

**A:** Selection of research reports for a methodological study depends on the research question and eligibility criteria. Once a clear research question is set and the nature of literature one desires to review is known, one can then begin the selection process. Selection may begin with a broad search, especially if the eligibility criteria are not apparent. For example, a methodological study of Cochrane Reviews of HIV would not require a complex search as all eligible studies can easily be retrieved from the Cochrane Library after checking a few boxes [30]. On the other hand, a methodological study of subgroup analyses in trials of gastrointestinal oncology would require a search to find such trials, and further screening to identify trials that conducted a subgroup analysis [31].

The strategies used for identifying participants in observational studies can apply here. One may use a systematic search to identify all eligible studies. If the number of eligible studies is unmanageable, a random sample of articles can be expected to provide comparable results if it is sufficiently large [32]. For example, Wilson et al. used a random sample of trials from the Cochrane Stroke Group’s Trial Register to investigate completeness of reporting [33]. It is possible that a simple random sample would lead to underrepresentation of units (i.e. research reports) that are smaller in number. This is relevant if the investigators wish to compare multiple groups but have too few units in one group. In this case a stratified sample would help to create equal groups. For example, in a methodological study comparing Cochrane and non-Cochrane reviews, Kahale et al. drew random samples from both groups [34]. Alternatively, systematic or purposeful sampling strategies can be used and we encourage researchers to justify their selected approaches based on the study objective.

**Q: How many databases should I search?**

**A:** The number of databases one should search would depend on the approach to sampling, which can include targeting the entire “population” of interest or a sample of that population. If you are interested in including the entire target population for your research question, or drawing a random or systematic sample from it, then a comprehensive and exhaustive search for relevant articles is required. In this case, we recommend using systematic approaches for searching electronic databases (i.e. at least 2 databases with a replicable and time stamped search strategy). The results of your search will constitute a sampling frame from which eligible studies can be drawn.

Alternatively, if your approach to sampling is purposeful, then we recommend targeting the database(s) or data sources (e.g. journals, registries) that include the information you need. For example, if you are conducting a methodological study of high impact journals in plastic surgery and they are all indexed in PubMed, you likely do not need to search any other databases. You may also have a comprehensive list of all journals of interest and can approach your search using the journal names in your database search (or by accessing the journal archives directly from the journal’s website). Even though one could also search journals’ web pages directly, using a database such as PubMed has multiple advantages, such as the use of filters, so the search can be narrowed down to a certain period, or study types of interest. Furthermore, individual journals’ web sites may have different search functionalities, which do not necessarily yield a consistent output.

**Q: Should I publish a protocol for my methodological study?**

**A:** A protocol is a description of intended research methods. Currently, only protocols for clinical trials require registration [35]. Protocols for systematic reviews are encouraged but no formal recommendation exists. The scientific community welcomes the publication of protocols because they help protect against selective outcome reporting, the use of post hoc methodologies to embellish results, and to help avoid duplication of efforts [36]. While the latter two risks exist in methodological research, the negative consequences may be substantially less than for clinical outcomes. In a sample of 31 methodological studies, 7 (22.6%) referenced a published protocol [9]. In the Cochrane Library, there are 15 protocols for methodological reviews (21 July 2020). This suggests that publishing protocols for methodological studies is not uncommon.

Authors can consider publishing their study protocol in a scholarly journal as a manuscript. Advantages of such publication include obtaining peer-review feedback about the planned study, and easy retrieval by searching databases such as PubMed. The disadvantages in trying to publish protocols includes delays associated with manuscript handling and peer review, as well as costs, as few journals publish study protocols, and those journals mostly charge article-processing fees [37]. Authors who would like to make their protocol publicly available without publishing it in scholarly journals, could deposit their study protocols in publicly available repositories, such as the Open Science Framework (https://osf.io/).

**Q: How to appraise the quality of a methodological study?**

**A:** To date, there is no published tool for appraising the risk of bias in a methodological study, but in principle, a methodological study could be considered as a type of observational study. Therefore, during conduct or appraisal, care should be taken to avoid the biases common in observational studies [38]. These biases include selection bias, comparability of groups, and ascertainment of exposure or outcome. In other words, to generate a representative sample, a comprehensive reproducible search may be necessary to build a sampling frame. Additionally, random sampling may be necessary to ensure that all the included research reports have the same probability of being selected, and the screening and selection processes should be transparent and reproducible. To ensure that the groups compared are similar in all characteristics, matching, random sampling or stratified sampling can be used. Statistical adjustments for between-group differences can also be applied at the analysis stage. Finally, duplicate data extraction can reduce errors in assessment of exposures or outcomes.

**Q: Should I justify a sample size?**

**A:** In all instances where one is not using the target population (i.e. the group to which inferences from the research report are directed) [39], a sample size justification is good practice. The sample size justification may take the form of a description of what is expected to be achieved with the number of articles selected, or a formal sample size estimation that outlines the number of articles required to answer the research question with a certain precision and power. Sample size justifications in methodological studies are reasonable in the following instances:

- Comparing two groups
- Determining a proportion, mean or another quantifier
- Determining factors associated with an outcome using regression-based analyses

For example, El Dib et al. computed a sample size requirement for a methodological study of diagnostic strategies in randomized trials, based on a confidence interval approach [40].

**Q: What should I call my study?**

**A:** Other terms which have been used to describe/label methodological studies include “*methodological review*”, *“methodological survey”*, *“meta-epidemiological study”*, *“systematic review”*, *“systematic survey”, “meta-research”, “research-on-research”* and many others. We recommend that the study nomenclature be clear, unambiguous, informative and allow for appropriate indexing. Methodological study nomenclature that should be avoided includes “*systematic review”* – as this will likely be confused with a systematic review of a clinical question. “*Systematic survey”* may also lead to confusion about whether the survey was *systematic* (i.e. using a preplanned methodology) or a survey using “*systematic”* sampling (i.e. a sampling approach using specific intervals to determine who is selected) [32]. Any of the above meanings of the words “*systematic”* may be true for methodological studies and could be potentially misleading. “*Meta-epidemiological study”* is ideal for indexing, but not very informative as it describes an entire field. The term “*review*” may point towards an appraisal or “review” of the design, conduct, analysis or reporting (or methodological components) of the targeted research reports, yet it has also been used to describe narrative reviews [41, 42]. The term “*survey*” is also in line with the approaches used in many methodological studies [9], and would be indicative of the sampling procedures of this study design. However, in the absence of guidelines on nomenclature, the term “*methodological study*” is broad enough to capture most of the scenarios of such studies.

**Q: Should I account for clustering in my methodological study?**

**A:** Data from methodological studies are often clustered. For example, articles coming from a specific source may have different reporting standards (e.g. the Cochrane Library). Articles within the same journal may be similar due to editorial practices and policies, reporting requirements and endorsement of guidelines. There is emerging evidence that these are real concerns that should be accounted for in analyses [43]. Some cluster variables are described in the section: “**What variables are relevant to methodological studies?”**

A variety of modelling approaches can be used to account for correlated data, including the use of marginal, fixed or mixed effects regression models with appropriate computation of standard errors [44]. For example, Kosa et al. used generalized estimation equations to account for correlation of articles within journals [15]. Not accounting for clustering could lead to incorrect *p*-values, unduly narrow confidence intervals, and biased estimates [45].

**Q: Should I extract data in duplicate?**

A: Yes. Duplicate data extraction takes more time but results in less errors [19]. Data extraction errors in turn affect the effect estimate [46], and therefore should be mitigated. Duplicate data extraction should be considered in the absence of other approaches to minimize extraction errors. However, much like systematic reviews, this area will likely see rapid new advances with machine learning and natural language processing technologies to support researchers with screening and data extraction [47, 48]. However, experience plays an important role in the quality of extracted data and inexperienced extractors should be paired with experienced extractors [46, 49].

**Q: Should I assess the risk of bias of research reports included in my methodological study?**

**A**: Risk of bias is most useful in determining the certainty that can be placed in the effect measure from a study. In methodological studies, risk of bias may not serve the purpose of determining the trustworthiness of results, as effect measures are often not the primary goal of methodological studies. Determining risk of bias in methodological studies is likely a practice borrowed from systematic review methodology, but whose intrinsic value is not obvious in methodological studies. When it is part of the research question, investigators often focus on one aspect of risk of bias. For example, Speich investigated how blinding was reported in surgical trials [50], and Abraha et al., investigated the application of intention-to-treat analyses in systematic reviews and trials [51].

**Q: What variables are relevant to methodological studies?**

A: There is empirical evidence that certain variables may inform the findings in a methodological study. We outline some of these and provide a brief overview below:

- Country: Countries and regions differ in their research cultures, and the resources available to conduct research. Therefore, it is reasonable to believe that there may be differences in methodological features across countries. Methodological studies have reported loco-regional differences in reporting quality [52, 53]. This may also be related to challenges non-English speakers face in publishing papers in English.
- Authors’ expertise: The inclusion of authors with expertise in research methodology, biostatistics, and scientific writing is likely to influence the end-product. Oltean et al. found that among randomized trials in orthopaedic surgery, the use of analyses that accounted for clustering was more likely when specialists (e.g. statistician, epidemiologist or clinical trials methodologist) were included on the study team [54]. Fleming et al. found that including methodologists in the review team was associated with appropriate use of reporting guidelines [55].
- Source of funding and conflicts of interest: Some studies have found that funded studies report better [56, 57], while others do not [53, 58]. The presence of funding would indicate the availability of resources deployed to ensure optimal design, conduct, analysis and reporting. However, the source of funding may introduce conflicts of interest and warrant assessment. For example, Kaiser et al. investigated the effect of industry funding on obesity or nutrition randomized trials and found that reporting quality was similar [59]. Thomas et al. looked at reporting quality of long-term weight loss trials and found that industry funded studies were better [60]. Kan et al. examined the association between industry funding and “positive trials” (trials reporting a significant intervention effect) and found that industry funding was highly predictive of a positive trial [61]. This finding is similar to that of a recent Cochrane Methodology Review by Hansen et al. [62]
- Journal characteristics: Certain journals’ characteristics may influence the study design, analysis or reporting. Characteristics such as journal endorsement of guidelines [63, 64], and Journal Impact Factor (JIF) have been shown to be associated with reporting [63, 65–67].
- Study size (sample size/number of sites): Some studies have shown that reporting is better in larger studies [53, 56, 58].
- Year of publication: It is reasonable to assume that design, conduct, analysis and reporting of research will change over time. Many studies have demonstrated improvements in reporting over time or after the publication of reporting guidelines [68, 69].
- Type of intervention: In a methodological study of reporting quality of weight loss intervention studies, Thabane et al. found that trials of pharmacologic interventions were reported better than trials of non-pharmacologic interventions [70].
- Interactions between variables: Complex interactions between the previously listed variables are possible. High income countries with more resources may be more likely to conduct larger studies and incorporate a variety of experts. Authors in certain countries may prefer certain journals, and journal endorsement of guidelines and editorial policies may change over time.

**Q: Should I focus only on high impact journals?**

**A:** Investigators may choose to investigate only high impact journals because they are more likely to influence practice and policy, or because they assume that methodological standards would be higher. However, the JIF may severely limit the scope of articles included and may skew the sample towards articles with positive findings. The generalizability and applicability of findings from a handful of journals must be examined carefully, especially since the JIF varies over time. Even among journals that are all “high impact”, variations exist in methodological standards.

**Q: Can I conduct a methodological study of qualitative research?**

**A:** Yes. Even though a lot of methodological research has been conducted in the quantitative research field, methodological studies of qualitative studies are feasible. Certain databases that catalogue qualitative research including the Cumulative Index to Nursing & Allied Health Literature (CINAHL) have defined subject headings that are specific to methodological research (e.g. “research methodology”). Alternatively, one could also conduct a qualitative methodological review; that is, use qualitative approaches to synthesize methodological issues in qualitative studies.

**Q: What reporting guidelines should I use for my methodological study?**

**A:** There is no guideline that covers the entire scope of methodological studies. One adaptation of the PRISMA guidelines has been published, which works well for studies that aim to use the entire target population of research reports [71]. However, it is not widely used (40 citations in 2 years as of 09 December 2019), and methodological studies that are designed as cross-sectional or before-after studies require a more fit-for purpose guideline. A more encompassing reporting guideline for a broad range of methodological studies is currently under development [72]. However, in the absence of formal guidance, the requirements for scientific reporting should be respected, and authors of methodological studies should focus on transparency and reproducibility.

**Q: What are the potential threats to validity and how can I avoid them?**

**A:** Methodological studies may be compromised by a lack of internal or external validity. The main threats to internal validity in methodological studies are selection and confounding bias. Investigators must ensure that the methods used to select articles does not make them differ systematically from the set of articles to which they would like to make inferences. For example, attempting to make extrapolations to all journals after analyzing high-impact journals would be misleading.

Many factors (confounders) may distort the association between the exposure and outcome if the included research reports differ with respect to these factors [73]. For example, when examining the association between source of funding and completeness of reporting, it may be necessary to account for journals that endorse the guidelines. Confounding bias can be addressed by restriction, matching and statistical adjustment [73]. Restriction appears to be the method of choice for many investigators who choose to include only high impact journals or articles in a specific field. For example, Knol et al. examined the reporting of *p*-values in baseline tables of high impact journals [26]. Matching is also sometimes used. In the methodological study of non-randomized interventional studies of elective ventral hernia repair, Parker et al. matched prospective studies with retrospective studies and compared reporting standards [74]. Some other methodological studies use statistical adjustments. For example, Zhang et al. used regression techniques to determine the factors associated with missing participant data in trials [16].

With regard to external validity, researchers interested in conducting methodological studies must consider how generalizable or applicable their findings are. This should tie in closely with the research question and should be explicit. For example. Findings from methodological studies on trials published in high impact cardiology journals cannot be assumed to be applicable to trials in other fields. However, investigators must ensure that their sample truly represents the target sample either by a) conducting a comprehensive and exhaustive search, or b) using an appropriate and justified, randomly selected sample of research reports.

Even applicability to high impact journals may vary based on the investigators’ definition, and over time. For example, for high impact journals in the field of general medicine, Bouwmeester et al. included the Annals of Internal Medicine (AIM), BMJ, the Journal of the American Medical Association (JAMA), Lancet, the New England Journal of Medicine (NEJM), and PLoS Medicine (*n* = 6) [75]. In contrast, the high impact journals selected in the methodological study by Schiller et al. were BMJ, JAMA, Lancet, and NEJM (*n* = 4) [76]. Another methodological study by Kosa et al. included AIM, BMJ, JAMA, Lancet and NEJM (*n* = 5). In the methodological study by Thabut et al., journals with a JIF greater than 5 were considered to be high impact. Riado Minguez et al. used first quartile journals in the Journal Citation Reports (JCR) for a specific year to determine “high impact” [77]. Ultimately, the definition of high impact will be based on the number of journals the investigators are willing to include, the year of impact and the JIF cut-off [78]. We acknowledge that the term “generalizability” may apply differently for methodological studies, especially when in many instances it is possible to include the entire target population in the sample studied.

Finally, methodological studies are not exempt from information bias which may stem from discrepancies in the included research reports [79], errors in data extraction, or inappropriate interpretation of the information extracted. Likewise, publication bias may also be a concern in methodological studies, but such concepts have not yet been explored.

### A proposed framework

In order to inform discussions about methodological studies, the development of guidance for what should be reported, we have outlined some key features of methodological studies that can be used to classify them. For each of the categories outlined below, we provide an example. In our experience, the choice of approach to completing a methodological study can be informed by asking the following four questions:

1. **What is the aim?**

- Methodological studies that investigate bias
  A methodological study may be focused on exploring sources of bias in primary or secondary studies (meta-bias), or how bias is analyzed. We have taken care to distinguish bias (i.e. systematic deviations from the truth irrespective of the source) from reporting quality or completeness (i.e. not adhering to a specific reporting guideline or norm). An example of where this distinction would be important is in the case of a randomized trial with no blinding. This study (depending on the nature of the intervention) would be at risk of performance bias. However, if the authors report that their study was not blinded, they would have reported adequately. In fact, some methodological studies attempt to capture both “quality of conduct” and “quality of reporting”, such as Richie et al., who reported on the risk of bias in randomized trials of pharmacy practice interventions [80]. Babic et al. investigated how risk of bias was used to inform sensitivity analyses in Cochrane reviews [81]. Further, biases related to choice of outcomes can also be explored. For example, Tan et al investigated differences in treatment effect size based on the outcome reported [82].
- Methodological studies that investigate quality (or completeness) of reporting
  Methodological studies may report quality of reporting against a reporting checklist (i.e. adherence to guidelines) or against expected norms. For example, Croituro et al. report on the quality of reporting in systematic reviews published in dermatology journals based on their adherence to the PRISMA statement [83], and Khan et al. described the quality of reporting of harms in randomized controlled trials published in high impact cardiovascular journals based on the CONSORT extension for harms [84]. Other methodological studies investigate reporting of certain features of interest that may not be part of formally published checklists or guidelines. For example, Mbuagbaw et al. described how often the implications for research are elaborated using the Evidence, Participants, Intervention, Comparison, Outcome, Timeframe (EPICOT) format [30].
- Methodological studies that investigate the consistency of reporting
  Sometimes investigators may be interested in how consistent reports of the same research are, as it is expected that there should be consistency between: conference abstracts and published manuscripts; manuscript abstracts and manuscript main text; and trial registration and published manuscript. For example, Rosmarakis et al. investigated consistency between conference abstracts and full text manuscripts [85].
- Methodological studies that investigate factors associated with reporting
  In addition to identifying issues with reporting in primary and secondary studies, authors of methodological studies may be interested in determining the factors that are associated with certain reporting practices. Many methodological studies incorporate this, albeit as a secondary outcome. For example, Farrokhyar et al. investigated the factors associated with reporting quality in randomized trials of coronary artery bypass grafting surgery [53].
- Methodological studies that investigate methods
  Methodological studies may also be used to describe methods or compare methods, and the factors associated with methods. Muller et al. described the methods used for systematic reviews and meta-analyses of observational studies [86].
- Methodological studies that summarize other methodological studies
  Some methodological studies synthesize results from other methodological studies. For example, Li et al. conducted a scoping review of methodological reviews that investigated consistency between full text and abstracts in primary biomedical research [87].
- Methodological studies that investigate nomenclature and terminology
  Some methodological studies may investigate the use of names and terms in health research. For example, Martinic et al. investigated the definitions of systematic reviews used in overviews of systematic reviews (OSRs), meta-epidemiological studies and epidemiology textbooks [88].
- Other types of methodological studies
  In addition to the previously mentioned experimental methodological studies, there may exist other types of methodological studies not captured here.

2. **What is the design?**

- Methodological studies that are descriptive
  Most methodological studies are purely descriptive and report their findings as counts (percent) and means (standard deviation) or medians (interquartile range). For example, Mbuagbaw et al. described the reporting of research recommendations in Cochrane HIV systematic reviews [30]. Gohari et al. described the quality of reporting of randomized trials in diabetes in Iran [12].
- Methodological studies that are analytical
  Some methodological studies are analytical wherein “analytical studies identify and quantify associations, test hypotheses, identify causes and determine whether an association exists between variables, such as between an exposure and a disease.” [89] In the case of methodological studies all these investigations are possible. For example, Kosa et al. investigated the association between agreement in primary outcome from trial registry to published manuscript and study covariates. They found that larger and more recent studies were more likely to have agreement [15]. Tricco et al. compared the conclusion statements from Cochrane and non-Cochrane systematic reviews with a meta-analysis of the primary outcome and found that non-Cochrane reviews were more likely to report positive findings. These results are a test of the null hypothesis that the proportions of Cochrane and non-Cochrane reviews that report positive results are equal [90].

3. **What is the sampling strategy?**

- Methodological studies that include the target population
  Methodological reviews with narrow research questions may be able to include the entire target population. For example, in the methodological study of Cochrane HIV systematic reviews, Mbuagbaw et al. included all of the available studies (*n* = 103) [30].
- Methodological studies that include a sample of the target population
  Many methodological studies use random samples of the target population [33, 91, 92]. Alternatively, purposeful sampling may be used, limiting the sample to a subset of research-related reports published within a certain time period, or in journals with a certain ranking or on a topic. Systematic sampling can also be used when random sampling may be challenging to implement.

4. **What is the unit of analysis?**

- Methodological studies with a research report as the unit of analysis
  Many methodological studies use a research report (e.g. full manuscript of study, abstract portion of the study) as the unit of analysis, and inferences can be made at the study-level. However, both published and unpublished research-related reports can be studied. These may include articles, conference abstracts, registry entries etc.
- Methodological studies with a design, analysis or reporting item as the unit of analysis
  Some methodological studies report on items which may occur more than once per article. For example, Paquette et al. report on subgroup analyses in Cochrane reviews of atrial fibrillation in which 17 systematic reviews planned 56 subgroup analyses [93].

**Fig. 2:**
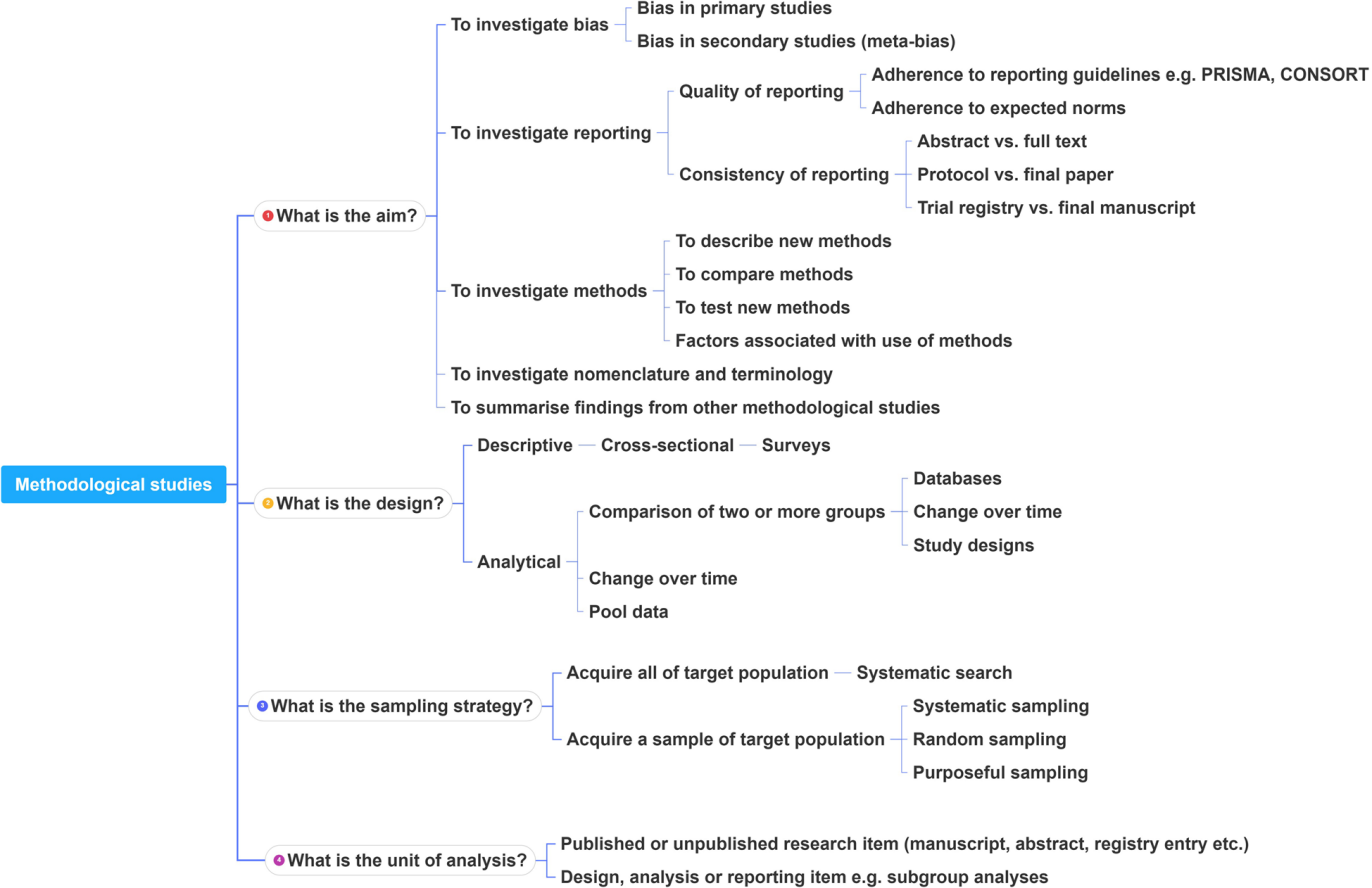
A proposed framework for methodological studies

This framework is outlined in Fig. 2.

## Conclusions

Methodological studies have examined different aspects of reporting such as quality, completeness, consistency and adherence to reporting guidelines. As such, many of the methodological study examples cited in this tutorial are related to reporting. However, as an evolving field, the scope of research questions that can be addressed by methodological studies is expected to increase.

In this paper we have outlined the scope and purpose of methodological studies, along with examples of instances in which various approaches have been used. In the absence of formal guidance on the design, conduct, analysis and reporting of methodological studies, we have provided some advice to help make methodological studies consistent. This advice is grounded in good contemporary scientific practice. Generally, the research question should tie in with the sampling approach and planned analysis. We have also highlighted the variables that may inform findings from methodological studies. Lastly, we have provided suggestions for ways in which authors can categorize their methodological studies to inform their design and analysis.

## Data Availability

Data sharing is not applicable to this article as no new data were created or analyzed in this study.

## Abbreviations

CONSORT: Consolidated Standards of Reporting Trials
EPICOT: Evidence, Participants, Intervention, Comparison, Outcome, Timeframe
GRADE: Grading of Recommendations, Assessment, Development and Evaluations
PICOT: Participants, Intervention, Comparison, Outcome, Timeframe
PRISMA: Preferred Reporting Items of Systematic reviews and Meta-Analyses
SWAR: Studies Within a Review
SWAT: Studies Within a Trial
